# Frailty is a stronger predictor of death in younger intensive care patients than in older patients: a prospective observational study

**DOI:** 10.1186/s13613-022-01098-2

**Published:** 2022-12-31

**Authors:** Lina De Geer, Mats Fredrikson, Michelle S. Chew

**Affiliations:** 1 Department of Anaesthesiology and Intensive Care, and Department of Biomedical and Clinical Sciences, Linköping University, 581 83 Linköping, Sweden; 2 Division of Occupational and Environmental Medicine, Department of Clinical and Experimental Medicine and Forum Östergötland, All at Linköping University, 581 83 Linköping, Sweden

**Keywords:** Frailty, Critical illness, Intensive care unit, Outcome

## Abstract

**Background:**

While frailty is a known predictor of adverse outcomes in older patients, its effect in younger populations is unknown. This prospective observational study was conducted in a tertiary-level mixed ICU to assess the impact of frailty on long-term survival in intensive care patients of different ages.

**Methods:**

Data on premorbid frailty (Clinical Frailty Score; CFS), severity of illness (the Simplified Acute Physiology Score, third version; SAPS3), limitations of care and outcome were collected in 817 adult ICU patients. Hazard ratios (HR) for death within 180 days after ICU admission were calculated. Unadjusted and adjusted analyses were used to evaluate the association of frailty with outcome in different age groups.

**Results:**

Patients were classified into predefined age groups (18–49 years (*n* = 241), 50–64 (*n* = 188), 65–79 (*n* = 311) and 80 years or older (*n* = 77)). The proportion of frail (CFS ≥ 5) patients was 41% (*n* = 333) in the overall population and increased with each age strata (*n* = 46 (19%) vs. *n* = 67 (36%) vs. *n* = 174 (56%) vs. *n* = 46 (60%), *P* < 0.05). Frail patients had higher SAPS3, more treatment restrictions and higher ICU mortality. Frailty was associated with an increased risk of 180-day mortality in all age groups (HR 5.7 (95% CI 2.8–11.4), *P* < 0.05; 8.0 (4.0–16.2), *P* < 0.05; 4.1 (2.2–6.6), *P* < 0.05; 2.4 (1.1–5.0), *P* = 0.02). The effect remained significant after adjustment for SAPS3, comorbidity and limitations of treatment only in patients aged 50–64 (2.1 (1.1–3.1), *P* < 0.05).

**Conclusions:**

Premorbid frailty is common in ICU patients of all ages and was found in 55% of patients aged under 64 years. Frailty was independently associated with mortality only among middle-aged patients, where the risk of death was increased twofold. Our study supports the use of frailty assessment in identifying younger ICU patients at a higher risk of death.

**Supplementary Information:**

The online version contains supplementary material available at 10.1186/s13613-022-01098-2.

## Key points

- A considerable proportion of younger patients requiring intensive care are frail.
- Frailty increases the risk of death up to 6 months after intensive care.
- The most marked effect of frailty on the risk of death is in middle-aged patients.

**Supplementary Information:**

The online version contains supplementary material available at 10.1186/s13613-022-01098-2.

## Background

The interest in frailty in intensive care unit (ICU) patients has grown rapidly in the last decade. Originally developed as a medical concept in geriatrics, the first applications of frailty assessment in intensive care medicine were in older patients [1, 2]. Numerous studies have since then demonstrated that frailty increases the risk of a wide range of negative outcomes in older ICU patients. More specifically, frailty increases the risk of a prolonged length of stay and of complications during intensive care, as well as death in older patients [2–8]. While its prevalence increases with age, frailty is a multidimensional syndrome indicating an increased vulnerability to stressors that is not limited to the elderly [9–11]. Often described as a reflection of a patients biological rather than chronologic age, the interest in frailty in younger patients is growing in intensive care medicine as well as in other medical fields [9, 12, 13]. Importantly, frailty contributes to adverse outcomes in critically ill patients independently of traditional risk factors, such as age, comorbidities and severity of illness, and is thought to reflect a dimension of health that is not measurable using current risk scoring systems [12]. Yet a recent rapid evidence review identified no evidence about the impact of measuring frailty in younger populations on patient outcomes [13]. This is an important knowledge gap because the impact of frailty in younger populations may be different than older adults.

This study aimed to assess the prevalence of frailty in an unselected group of general ICU patients, and its impact on long-term survival in different age groups. Our hypothesis was that frailty was an independent predictor of a negative outcome in intensive care patients even among younger patients.

## Methods

This study was approved by the Regional Ethical Review Board in Linköping, Sweden (Dnr 2016/537-31). Due to the observational nature of the study, the requirement for informed consent was waived. A formal license agreement with permission to use the Clinical Frailty Scale (CFS) was obtained from the copyright holder (Rockwood K. Permission for use agreement. 2018.), and the study was conducted in accordance with the STROBE statement [14].

Adult patients admitted to a mixed, non-cardiothoracic, tertiary general ICU in a university hospital, from January 2017 to June 2018, were included in the study. Patients were included only once, and patients with multiple admissions were included only as per their primary admission. Data on patient characteristics such as age and comorbidities, admission details and severity of illness using the Simplified Acute Physiology Score (SAPS3) were collected, in addition to data on treatment received in the ICU, on whether treatment was withheld or withdrawn, on complications during ICU stay and on ICU length of stay, as well as data on mortality.

The level of frailty in individual patients was defined as that preceding the acute illness and hospital admission, and the Clinical Frailty Scale (CFS) was used as a categorization tool. Information necessary for the assessment was collected from the patient, members of family and from patient charts, after admission to the ICU. The assessment was performed by one of the treating physicians in the ICU, who were all trained in the use of CFS before the start of the study [15]. Patients were categorized as non-frail or frail using a cut-off point as previously described [12], and all patients were classified in predefined age groups.

The primary outcome measure of this study was all-cause mortality within 180 days of ICU admission. Secondary outcome measures were complications and length of stay in the ICU.

### Statistical analysis

Data are presented as medians (lower to upper quartiles) and numbers (percentages). Groups of patients were compared regarding baseline characteristics, treatments and outcomes using the Mann–Whitney U-test and the Chi-square test, whereas the ANOVA test was used for the comparison of multiple groups. Multivariable Cox regression analyses were used to evaluate the independent effect of frailty on 180-day mortality with adjustment for comorbidities, SAPS3 score, and decision to withhold or withdraw therapy. To assess the effect of frailty in each age group, we repeated the analysis for each age strata using comorbidities, decision to withhold or withdraw therapy and frailty as independent factors. Previous audit data indicated that ICU mortality was approximately 20% in our population. Therefore, using a rule of thumb of 10 outcomes for each adjusted factor we evaluated the sample size as adequate to provide a robust multivariable analysis. A *P* < 0.05 was considered statistically significant for all comparisons, and all statistical analyses were performed using IBM SPSS 25.0 (IBM Corp, Armonk, NY, USA).

## Results

There were 1181 admissions to the ICU during the study period. Children (*n* = 172) and patients lost to follow-up (*n* = 55) were excluded, and patients admitted more than once during the study period (*n* = 127) were included only as per their primary admission. A total of 817 individual patients were included in the study (Additional file 1). The median (IQR) age was 63 (44–73) years, 476 (58%) of the patients were male, and 747 (91%) were admitted to the ICU for non-elective reasons. At the time of ICU admission, the median (IQR) SAPS3 score was 56 (43–67), and similar proportions of patients were admitted from the emergency department (*n* = 225; 28%), hospital wards (*n* = 232; 28%) and from the operating theatre or the postoperative high dependency unit (*n* = 193; 24%). The median (IQR) length of stay in the ICU was 26 (13–70) hours. During that time, 432 (53%) of the patients were on invasive mechanical ventilation and 40 (5%) on continuous renal replacement therapy. The most common ICU diagnoses were sepsis or septic shock (*n* = 190; 23%), respiratory insufficiency (*n* = 117; 14%), cardiac arrest (*n* = 90; 11%) and multiple trauma (*n* = 88; 11%) (Additional file 2).

**Fig. 1:**
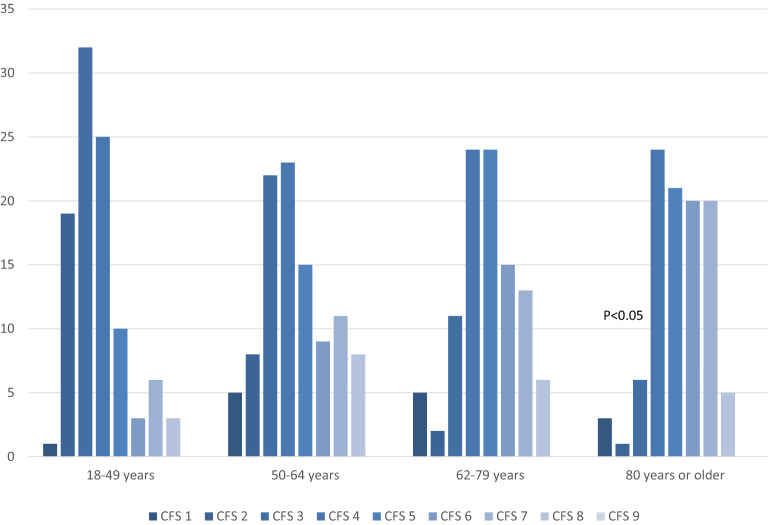
Clinical Frailty Scale (CFS) scores stratified by age groups

**Fig. 2:**
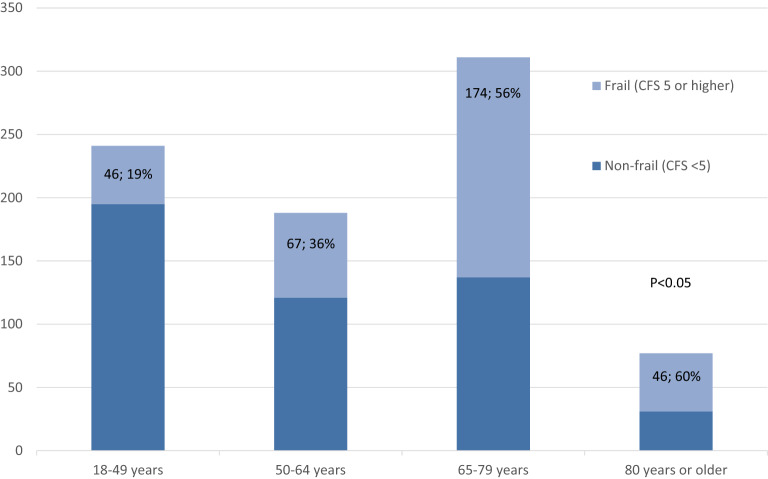
Proportions of frail (CFS ≥ 5, *n*; %) and non-frail (CFS < 5) patients in different age groups

**Fig. 3:**
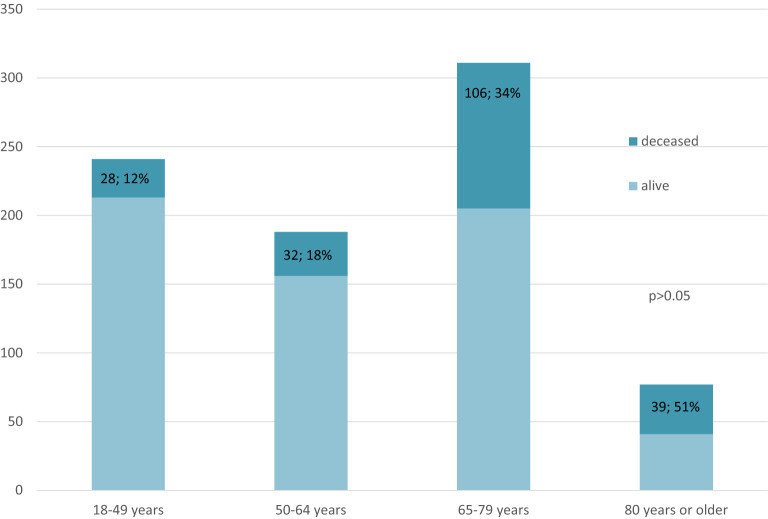
Mortality status 180 days after admission to the ICU in different age groups (*n*; %)

The median (IQR) CFS was 4 (3–6). The previously defined cut-off level regarding frailty of CFS 5 or higher [12] categorized 333 (41%) patients as frail. Patients were divided into four age groups [18–49 years (*n* = 241), 50–64 (*n* = 188), 65–79 (*n* = 311) and 80 years or older (*n* = 77)]. All levels of CFS were found in all age groups but in differing proportions (Fig. 1). Frail patients (CFS ≥ 5) were found in all age groups, but the proportion was larger with increasing age (Fig. 2) and the proportion of patients who died within 180 days of ICU admission was larger among older patients (Fig. 3). Among patients aged 64 and under, 55% were considered frail (Table 1). The incidence of complications during ICU care did not differ between frail and non-frail, whereas ICU length of stay was longer in frail patients in all age groups except the very old (> 80 years) (Table 1) (Additional file 3).

**Table 1:** Comparison of patients according to frailty regarding baseline demographics, clinical course, and outcome

	Non-frail patients (CFS (< 5)	Frail (CFS ≥ 5)	*P*
Patients aged 18–49 years, *n* = 241	195	46	
Sex, male	107 (55)	56 (57)	0.49
SAPS3	56 [47–66]	56 [47–66]	0.24
Comorbidities according to SAPS 3	26 (13)	15 (33)	0.003
Complications during ICU care	31 (16)	12 (26)	0.08
Withhold/withdraw decision in ICU	5 (3)	6 (13)	0.008
ICU length of stay, hours	18 [6–37]	27 [13–93]	0.02
Dead at 180 days	15 (8)	13 (28)	< 0.001
Patients aged 50–64 years *n* = 188	121	67	
Sex, male	66 (55)	46 (69)	0.04
SAPS3	49 [40–59]	58 [46–67]	0.000
Comorbidities according to SAPS3	34 (28)	19 (28)	0.55
Complications during ICU care	23 (19)	16 (24)	0.27
Withhold/withdraw decision in ICU	5 (4)	18 (27)	< 0.001
ICU length of stay, hours	22 [12–57]	44 [20–170]	< 0.001
Dead at 180 days	5 (4)	27 (40)	< 0.001
Patients aged 65–79 years *n* = 311	137	174	
Sex, male	86 (63)	101 (58)	0.233
SAPS3	59 [47–71]	66 [53–79]	< 0.001
Comorbidities according to SAPS3	35 (26)	64 (37)	0.02
Complications during ICU care	28 (20)	25 (14)	0.10
Withhold/withdraw decision in ICU	11 (8)	62 (36)	< 0.001
ICU length of stay, hours	24 [14–55]	45 [20–98]	< 0.001
Dead at 180 days	22 (16)	84 (48)	< 0.001
Patients aged 80 years or older *n* = 77	31	46	
Sex, male	22 (69)	22 (48)	0.04
SAPS3	68 [64–80]	67 [56–78]	0.85
Comorbidities according to SAPS3	8 (26)	8 (17)	0.27
Complications during ICU care	7 (23)	19 (22)	0.57
Withhold/withdraw decision in ICU	11 (36)	27 (59)	0.04
ICU length of stay, hours	23 [13–71]	23 [12–62]	0.86
Dead at 180 days	8 (26)	28 (61)	0.002

Data are presented as medians (IQR), numbers (*n*) and percentages (%) of patients

*CFS* Clinical Frailty Scale, *ICU* intensive care unit, *IQR* inter-quartile range, *SAPS3* Simplified Acute Physiology Score, third version

Frailty was associated with an increased hazard of 180-day mortality in all age groups (Table 2). Multivariable analysis with illness severity, comorbidities, decisions to withhold or withdraw therapy, and frailty demonstrated that frailty was independently associated with 180-day mortality among patients aged 50–64 but not in the other age groups (Table 2).

**Table 2:** Hazard ratios (HR (95% CI)) for death within 180 days of admission to the ICU in frail vs. non-frail patients

	Hazard ratio (95% CI)	*P*
Unadjusted		
Frail (CFS ≥ 5) vs. non-frail (CFS < 5), 18–49 years	5.7 (2.8–11.4)	< 0.05
Frail vs. non-frail, 50–64 years	8.0 (4.0–16.2)	< 0.05
Frail vs. non-frail, 65–79 years	4.1 (2.6–6.6)	< 0.05
Frail vs. non-frail, 80 years or older	2.4 (1.1–5.0)	0.02
Adjusted		
Frail vs. non-frail, 50–64 years	2.1 (1.4–3.1)	< 0.05

## Discussion

This study was conducted to study the impact of frailty on survival after intensive care in patients of different ages. We show that while frailty is common in critically ill patients of all ages, a large proportion of adults aged 64 years and under were considered frail. The negative impact of frailty in patients is more important in the non-elderly, more specifically in the middle-aged, where it was independently associated with a twofold increased hazard of 180-day mortality. Our study thereby provides new insights into the prevalence of frailty and its impact on the risk of death in younger critically ill patients.

While the concept of frailty as a medical syndrome was developed in geriatric medicine [1, 16], there has been a rapidly increasing interest in other specialties [17]. Most studies on frailty in the intensive care field have focused on older patients [3, 5, 7, 18] or on specific patient groups such as those with kidney injury, cardiac surgery or, more recently, COVID-19 [19–21]. Very few studies have focused on younger patients [7, 22] where it is plausible that frailty may even play a prognostic role independent of conventional severity of illness scores. In a recent, large study the implementation of routine frailty screening in intensive care patients of all ages in Australia and New Zealand was reported. This study also demonstrated the negative prognostic impact of frailty in younger patients [23]. Our study adds to the limited body of knowledge regarding the impact of frailty in intensive care patients of all ages, further supporting a broad approach to the use of frailty assessment in intensive care.

The proportion of frail patients was larger in all age groups in our study than in previous similar studies. In one large international study on intensive care patients aged 80 years or older [5], 40% of the patients were categorized as frail, markedly less than the 60% in our study. One study on frailty in middle-aged intensive care patients demonstrated frailty in 28% of the patients [22], and in the large binational study previously mentioned, 23% of patients aged 50 or older were considered frail [23]. Among younger patients the prevalence of frailty differed even more between studies, with 6% of patients younger than 50 considered frail in Australia and New Zealand, but 19% in our study [23]. Notably over half of patients aged 64 or under were considered frail in our cohort. Despite the larger proportion of frail patients in our study, mortality at 180 days or more from ICU admission did not differ markedly from previous comparable studies [6]. These differences and similarities may have been influenced by factors such as differences in healthcare organization in general, ICU thresholds and occupancy as well as the availability of ICU beds [5].

Unsurprisingly, the proportion of patients who had died within 180 days of being admitted to the ICU was lower in younger patients. Equally unsurprising, a larger proportion of frail patients were dead within 180 days in all age groups, all in line with previous studies [5, 7, 24]. An interesting finding, however, was that frailty remained an independent risk factor for mortality after adjustment for illness severity, comorbidities, and decisions to withhold or withdraw care, only in the group best described as middle-aged. In previous studies, the probability of survival for younger frail patients has appeared higher than for older patients [7], whereas our results coincide with the large study where frailty was an independent predictor of negative outcome, also in younger patients, after adjustment for severity of illness [23]. Indeed, since frailty is strongly correlated with age and often seen as an age-associated decline in physiological reserves and function [25, 26], it has been suggested to add frailty to the clinical assessment in elderly patients [5]. However, as shown here, frailty is relevant among younger critically ill patients [11, 22, 23]. It has indeed been suggested that the manifestation of frailty earlier in life conveys information about an accumulation of deficits that biological age and comorbidities alone does not [9]. Our study thereby adds to previous indications that frailty can be seen as a reflection of biological age, rather than chronological, and provides further support to the use of frailty assessment in intensive care patients of all ages.

Comorbidity and frailty are linked, but not completely synonymous [27, 28]. Although the prevalence of both comorbidity and frailty is higher in the elderly, there are indications that there is a greater overlap of the two in younger patients [7, 9, 27]. The comorbidities considered in this study, however, were limited to those included in SAPS3, all of which mirror relatively severe disease [29]. In fact, frailty seems to be more related to activities of daily life, which we have not studied. Nonetheless, our study indicates an independent value of frailty assessment in younger patients. Our results also support previous findings that frailty adds to conventional risk assessment scores [12, 23, 30, 31]. Thus, in outcome prediction, frailty mirrors a dimension not accounted for by comorbidities or by risk scores, especially in younger patients. This suggests that frailty assessment should be applied across the whole adult age span and, particularly in middle-aged ICU patients. Further research is warranted to determine how frailty may complement the clinician’s careful assessment of an individual’s functional and physiological reserve, multimorbidity and response to treatment, irrespective of age.

### Limitations and strengths

Our study has some limitations that must be considered. First, all patients were recruited following ICU admission to one single ICU. Differences in hospital characteristics, admission criteria and case-mix may therefore limit the generalizability of the study. Second, the identification of premorbid frailty in patients presenting in an intensive care setting may be hampered by factors related to the acute illness and events leading up to it, which may be difficult to separate from true frailty. We cannot exclude that the assessment may have been affected by events leading up to the admission to, and the patient’s status in, the intensive care unit. Third, sociodemographic factors and more detailed data on premorbid comorbidities were not available, all of which could have been used for statistical adjustment purposes. Importantly, we were not able to study interactions between individual comorbidities, concomitant diagnoses, and frailty. Last, other outcome measures than death, such as the risk of complications after ICU stay, return to home or previous level of independency in frail and non-frail patients, were not available. The lack of patient-related outcome measures such as health related quality of life is a notable limitation. Strength of the study lies in the inclusion of all patients presenting in a general ICU, without limitation to patients of a certain age or diagnosis, and in the overall number of patients included.

## Conclusion

Premorbid frailty is common in critically ill patients of all ages and affects a majority of patients under 64 years of age. The extent to which frailty is associated with an increased risk of death is most marked among middle-aged patients. We conclude that frailty needs to be recognized in all adult critically ill patients, especially the middle-aged.

## Supplementary Information

**Additional file 1. **Patient selection flow-chart.

**Additional file 2**. The nine-step Clinical Frailty Scale used for assessment of a patient’s degree of pre-existing frailty.

**Additional file 3. **Hazard ratios for death within 180 days of admission to the ICU for age, decision to withhold or withdraw therapy, SAPS3, presence of comorbidity and presence of frailty.

## Data Availability

The data sets used and analysed in the study are available from the corresponding author on reasonable request.

## Abbreviations

CFS: Clinical Frailty Scale
CI: Confidence interval
HR: Hazard ratio
ICU: Intensive care unit
IQR: Inter-quartile range
ROC-curve: Receiver operating characteristics curve
SAPS3: Simplified Acute Physiology Score, third version
