# Supplementary figures and images for: Frailty is a stronger predictor of death in younger intensive care patients than in older patients: a prospective observational study
Source: Ann Intensive Care. 2022 Dec 31;12:120. doi: 10.1186/s13613-022-01098-2 (PMC9803889; doi:10.1186/s13613-022-01098-2)

## Supplementary file 1

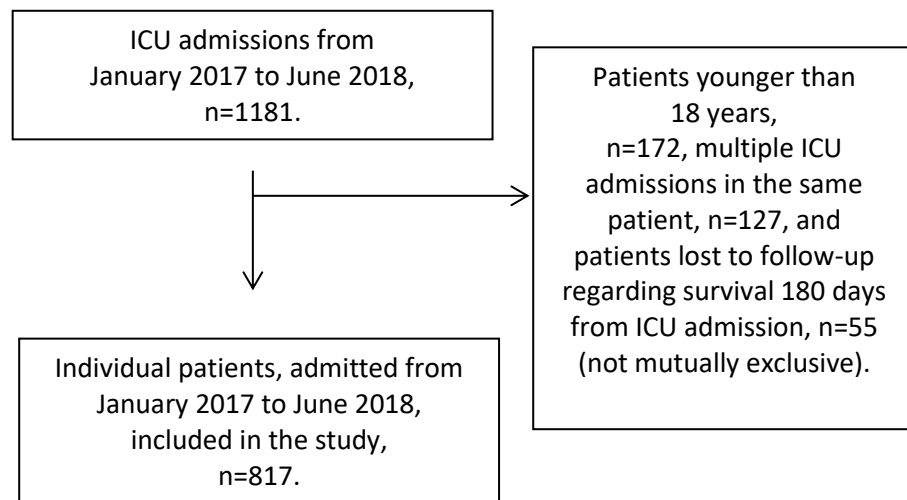

Supplement: Supplementary file 1 — Additional file 1. Patient selection flow-chart. [file 13613_2022_1098_MOESM1_ESM.pdf]
