# Supplementary material for: Frailty is a stronger predictor of death in younger intensive care patients than in older patients: a prospective observational study
Source: Ann Intensive Care. 2022 Dec 31;12:120. doi: 10.1186/s13613-022-01098-2 (PMC9803889; doi:10.1186/s13613-022-01098-2)
Supplement: Supplementary file 2 — Additional file 2. The nine-step Clinical Frailty Scale used for assessment of a patient’s degree of pre-existing frailty. [file 13613_2022_1098_MOESM2_ESM.pdf]

Score	Frailty grade	Description
1.	**Very fit**	People who are robust, active, energetic and motivated. These people commonly exercise and are the fittest for their age.
2.	**Well**	People who have no active disease symptoms but are less fit than those of category 1. Often, they exercise or are very active occasionally (that is, seasonally).
3.	**Managing well**	People whose medical problems are well controlled, but are not regularly active beyond routine walking.
4.	**Vulnerable**	While not dependent on other for daily help, symptoms often limit activities. A common complaint is being slowed up, and/or being tired during the day.
5.	**Mildly frail**	These people often have more evident slowing, and need help in high-order independent activities of daily living (finances, transportation, heavy housework, medications). Typically, mild frailty progressively impairs shopping and walking outside alone, meal preparation and housework.
6.	**Moderately frail**	People need help with all outside activities and with keeping house. Inside, they often have problems with stairs and need help with bathing and might need minimal assistance (cuing, standby) with dressing.
7.	**Severely frail**	Completely dependent for personal care, from whatever cause (physical or cognitive). Even so, they seem stable and not at a high risk of dying (within approximately 6 months).
8.	**Very severely frail**	Completely dependent, approaching the end of life. Typically, they could not recover even from a minor illness.
9.	**Terminally ill**	Approaching the end of life. This category applies to people with a life expectancy <6 months, who are not otherwise evidently frail.
