# Supplementary material for: Frailty is a stronger predictor of death in younger intensive care patients than in older patients: a prospective observational study
Source: Ann Intensive Care. 2022 Dec 31;12:120. doi: 10.1186/s13613-022-01098-2 (PMC9803889; doi:10.1186/s13613-022-01098-2)
Supplement: Supplementary file 3 — Additional file 3. Hazard ratios for death within 180 days of admission to the ICU for age, decision to withhold or withdraw therapy, SAPS3, presence of comorbidity and presence of frailty. [file 13613_2022_1098_MOESM3_ESM.pdf]

Additional file 3.

Hazard ratios (HR (95 % CI)) for death within 180 days of admission to the ICU. CFS: Clinical Frailty Scale; SAPS3: Simplified Acute Physiology Score, third version.

**Variable**	**Hazard ratio (95% CI)**	**P**
Age	1.0 (0.99 to 1.01)	0.93
Decision to withhold or withdraw therapy	4.8 (3.5 to 6.7)	<0.05
SAPS3	1.04 (1.0 to 1.1)	<0.05
Presence of comorbidity	1.4 (1.0 to 1.9)	0.04
Presence of frailty (CFS ≥5)	2.3 (1.4 to 3.3)	<0.05
